# Effective preparation of low-melting solder materials for atom probe tomography

**DOI:** 10.1038/s41598-024-79753-w

**Published:** 2024-11-27

**Authors:** Charlotte Cui, Michael Tkadletz, Michael Reisinger, Peter Imrich, Walter Hartner, Roland Brunner

**Affiliations:** 1 Materials Center Leoben Forschung GmbH, Roseggerstraße 12, 8700 Leoben, Austria; 2 Montanuniversität Leoben, Chair of Functional Materials at the Department Materials Science, Roseggerstraße 12, Leoben, 8700 Austria; 3 Kompetenzzentrum für Automobil- und Industrieelektronik GmbH, Europastraße 8, 9524 Villach, Austria; 4 Infineon Technologies AG, Wernerwerkstraße 2, 93049 Regensburg, Germany

**Keywords:** Materials science, Materials for devices, Techniques and instrumentation

## Abstract

Low-melting metal alloys have gained renewed attention for additive manufacturing, energy storage and microelectronics. However, micro- and nanostructure characterisation demands highly sophisticated sample preparation. Here, we optimise the Ga-FIB preparation of atom probe tomography (APT) specimens for low melting SAC305 solder materials utilising different FESEM/FIB stage temperatures. We study the effects of FESEM/FIB stage temperature on the specimen milling behaviour during Ga-FIB preparation and compare the extent of Ga implantation and precipitate coarsening during the preparation utilising energy dispersive X-ray spectroscopy and APT. We show that cooling the sample to −60 °C during FIB milling utilising a Peltier cooling stage improves the behaviour of the specimen during the final low-keV milling step significantly. We conclude that performing all Ga-FIB-sample interactions at −60 °C with a Pt-protection layer allows for effective and reproducible APT specimen preparation for low-melting alloys, such as SAC305.

## Introduction

Low-melting metal alloys, such as (near-) eutectic alloys, have gained renewed attention for additive manufacturing, namely liquid metal printing^1–3^, for liquid phase catalysts^1,4,5^as well as for energy storage media^1,6,7^. For all these applications, understanding the mechanical, electrical and thermal properties of low-melting metal alloys is indispensable. In order to understand the properties of materials, as well as their behaviour under load, the visualisation of their underlying micro- and nanostructures is essential. For many analyses on such small scales, e.g. utilising transmission electron microscopy (TEM) or atom probe tomography (APT), small specimen dimensions are required to provide electron transparency^8^and electric field enhancement^9,10^, respectively. To prepare these small specimens from a larger region of interest (ROI), a focussed ion beam (FIB) is commonly used with simultaneous scanning electron microscopy (SEM) for imaging^9,10^. However, targeted preparation of low-melting and metastable materials utilising FIB poses serious issues due to ion beam induced preparation artefacts^11–14^. Therein, the kinetic energy of the impinging ions is transferred to the sample as heat or momentum change^11^, which may cause ion implantation^15^, partial amorphization^16^, redeposition of sputtered material^17^and local heating^11^. The extent of local heating of the sample is dependent on its thermal conductivity and geometry, as well as the FIB parameters, like voltage, current and beam diameter^11^. Especially, for low-melting materials , this ion beam-induced local heating poses issues, as it may lead to significant microstructural changes^11,14^. Previous work has shown that the utilisation of a cryo-FIB with liquid nitrogen for the preparation of low-melting metals is essential^18^. Yet, cryo-FIBs are difficult to access and their utilisation is fairly elaborate, resource-intensive as well as time-consuming.

In the power- and microelectronics industry, reliable interconnects are vital for both electrical and thermal connection between chips and printed circuit boards (PCBs)^19–23^. Here, low-melting metals are widely utilised as solder materials, since the materials that are processed cannot be exposed to high temperatures as would arise during welding. Solders also allow the joining of non-melting materials. The differing wettability of different materials has given rise to a wide variety of solder materials with specific applications and their development is still ongoing. As the fabrication of these interconnects is one of the last production steps in the electronic industry, the devices that are being connected are usually already in their final shape. The precedingly fabricated chips, functionalised by, inter alia, multiple repetitions of selective etching and oxidation, are structurally extremely sensitive. Furthermore, subsequent chip packaging is often performed with polymers, hence the final interconnection of these electronic building blocks should be thermally as non-invasive as possible^24–26^. To that end, low-melting solder materials are specifically designed and continuously improved in order to accommodate the needs for low-temperature processing, high electrical and thermal conductivity, as well as mechanical integrity^24,27,28^. As these solders ensure the electrical and thermal connection between various electronic components in a device, their lifetime and intactness have a significant impact on the lifetime and functionality of the entire device^28,29^. Therefore, the understanding of their fatigue behaviour under load is vital. Low-melting Tin (Sn)-based solder alloys have largely replaced Lead-based alloys in power- and microelectronics due to growing health and environmental concerns^30,31^. As shown by Cui et al^32^., the predominant damage mechanism in Sn-based solder alloys during thermal cycling on board (TCoB) is intergranular fatigue crack propagation through the solder balls, preceded by dynamic recrystallisation of initially single- or few-grained balls. This conclusion is in keeping with other works^27,32–37^. Although recrystallised grain boundaries of the β-Sn matrix have been identified as weak-spots in the interconnect in the aforementioned studies, nano-scale investigation of these grain boundaries is scarce in literature. Some work has been previously done by utilising TEM^36,38^ but essential elemental analyses of recrystallised β-Sn grain boundaries are, to our knowledge, not yet published neither with TEM nor with APT since it demands a highly sophisticated preparation workflow of the specimen under investigation .

Therefore, the aim of this study is to optimise the gallium (Ga) -FIB preparation of APT-specimens for low-melting SAC305 solder materials. APT is utilised for the analysis of Ga-implantation during the specimen preparation at various stage temperatures. We study the effects of FESEM/FIB-stage temperature on the specimen milling behaviour during Ga-FIB preparation and compare the extent of Ga-implantation during the preparation utilising energy dispersive X-ray spectroscopy (EDX) and APT. We observe that FIB-milling at 25 °C results in Ga-implantation artefacts and precipitate coarsening in the lamella cross-sections, as well as over-heating artefacts during final low-keV milling of APT-specimens. Cooling the sample stage to -60 °C during FIB-milling utilising a Peltier cooling stage improves the behaviour of the sample during the final low-keV milling step significantly. At -60 °C, APT-specimens from low-melting SAC305 can be prepared using standard annular milling methods^9,10,39–41^, without over-heating artefacts. Furthermore, cooling the stage to -60 °C during trench cutting in the bulk and annular milling of the APT-specimens reduces Ga-implantation into the material significantly, compared to milling at 25 °C and −30°C. We illustrate, that the proposed preparation for APT utilising a Peltier cooling stage in the FESEM/FIB is proven to be a viable alternative to cryo-FIBs. Because the final radii of APT-specimens that are fabricated with a platinum (Pt-) protection layer are rather blunt, we substitute Pt with an aluminium (Al) -protection layer. We study the effects of the Al-protection layer on the specimen shape and Ga-implantation for the preparation at -60 °C. Despite the possibility to fabricate specimens with smaller initial radii compared to specimens with a Pt-protection layer, Al does not further reduce Ga-implantation. Therefore, we conclude that performing all Ga-FIB – specimen interactions at -60 °C allows for effective and reproducible APT-specimen preparation for low-melting alloys, such as SAC305.

## Results

### Microstructural, crystallographic and elemental information for the preparation of SAC305-samples

**Fig. 1:**
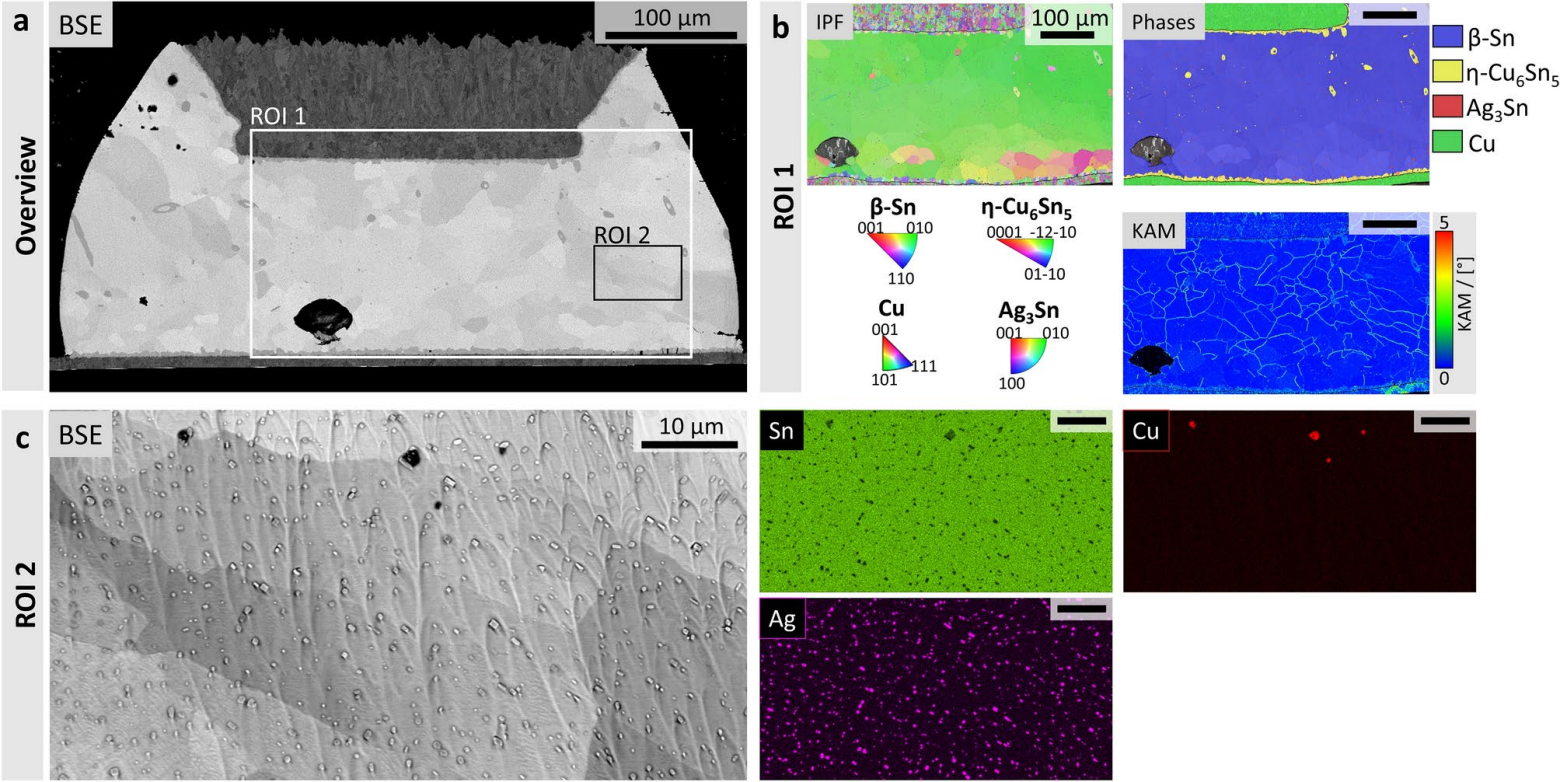
FESEM micrographs, EBSD-mappings and EDX-mappings of the investigated SAC305-sample**. a** FESEM-BSE overview micrograph of the cross-section. ROI 1 and ROI 2 are indicated with a white and black box, respectively. **b** EBSD-mappings of ROI 1, also indicated in **a**. In detail, IPF-, KAM- and phase-maps are shown for ROI 1. The indexed phases comprise β-Sn (blue), η-Cu_6_Sn_5_ (yellow), Cu (green) and Ag_3_Sn (red). Scale bar of 100 µm is valid for all images. **c** ROI 2 from **a** which is selected for the extraction of the lamellae. The FESEM-BSE micrograph is shown, alongside the EDX Sn-map in green, EDX Ag-map in magenta, EDX Cu-map in red for the respective L-shell energies. Scale bar of 10 µm is valid for all images.

In order to select a suitable region for the preparation of APT-specimens, the microstructure of the sample is visualised with a field emission SEM (FESEM). The sample production, as well as its cross-sectional preparation for FESEM imaging is described in Methods. Figure 1a shows the FESEM back-scatter electron (BSE) cross-sectional overview of the investigated sample. Figure 1b shows electron back-scatter diffraction (EBSD-) maps of the region of interest (ROI) labelled as ROI 1 in Fig. 1a. Further, the inverse pole figure (IPF), kernel average misorientation (KAM), as well as the phase-maps are shown. The IPF-map in Fig. 1b shows that the investigated solder ball consists of one large grain, as well as some smaller, dynamically recrystallised grains in its lower-right part. However, the KAM-map in Fig. 1b reveals some small contortions (~ 2°) within the large grain, which correspond to the grey-scale variations in channelling contrast in the BSE-micrograph of Fig. 1a. Lastly, the overview phase map in Fig. 1b reveals the matrix of the solder to be the β-Sn phase, whereas the metallisations are indexed as fcc-Cu. Moreover, are η-Cu_6_Sn_5_ particles not only shown to be embedded in the β-Sn matrix, but also is the η-Cu_6_Sn_5_ phase indexed at the Cu – Sn interfaces.The orthorhombic Ag_3_Sn phase is found to be finely dispersed in the β-Sn matrix. In order to prevent crystallographic artefacts in the milling behaviour and Ga-implantation, ROI 2, is chosen for the preparation of APT specimens. ROI 2 is located within a single crystal region, where there are no η-Cu_6_Sn_5_ particles present, as they may also influence the material behaviour locally, see Fig. 1a. The elemental composition of ROI 2 is mapped using EDX which is shown in Fig. 1c. Here, the Sn-matrix, as well as the Ag-rich Ag_3_Sn and Cu-rich Cu_6_Sn_5_ phases in ROI 2 can be distinguished. Details about BSE-, EBSD- and EDX-imaging and analysis are given in Methods.

### Investigation of the effect of stage temperature during Ga-FIB trench-milling on precipitate coarsening and Ga-implantation

**Fig. 2:**
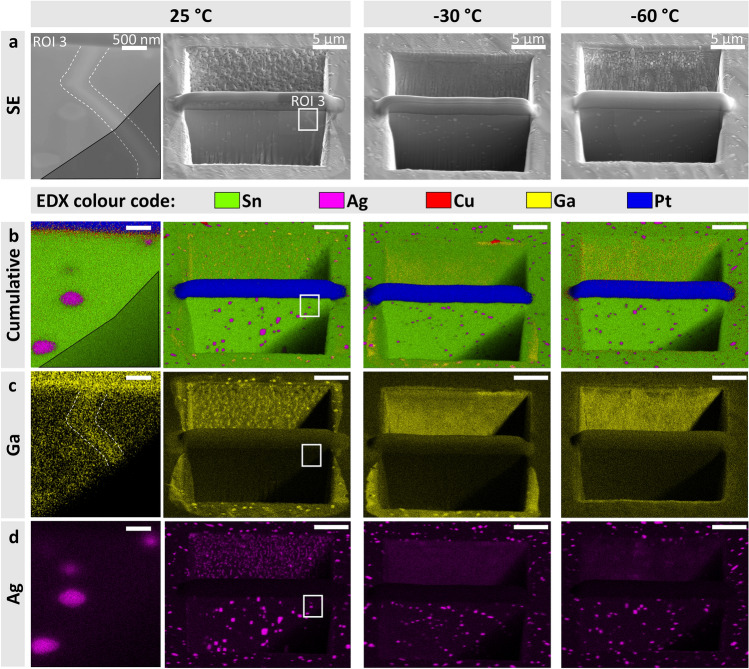
Trench milling and lamellae cross-sections for lift-outs at the investigated stage temperatures**.** FESEM-SE imaging and EDX-maps under 56° stage-tilt of the trenches that were cut at various sample stage-temperatures. From left to right: 25 °C, -30 °C, -60 °C. The first column shows the detail of ROI 3 that is marked in the 25 °C-trench with a white box. **a** SE-imaging of the trenches. **b** EDX-mappings of the respective SE-micrographs shown in **a**. The following elements are mapped: Sn (green), Ag (magenta), Cu (red), Ga (yellow) and Pt (blue). The area of ROI 3 that is shadowed by the trench side-wall during EDX-mapping is indicated by black triangles. **c** Separate Ga EDX-mappings of the respective SE-micrographs shown in **a**. Ga-enrichment can be seen in the 25 °C-lamella, which is marked with dashed lines in ROI 3. **d** Separate Ag EDX-mappings of the respective SE-micrographs shown in **a**. Scale bar of 500 nm and 5 µm as indicated in **a**, is valid for **b** and **c**.

For the preparation of APT-specimens, the lamellae need to be extracted from the bulk. In order to study the effect of stage temperature during Ga-FIB preparation on milling behaviour and Ga-implantation, three stage temperatures are investigated: 25, −30 and −60 °C. FESEM-secondary electron (SE) micrographs of the final lamellae are shown in Fig. 2a, alongside with EDX-mappings in Fig. 2b**–**d. A detail of the 25 °C-trench, indicated as ROI 3, is also shown. Figure 2b depicts cumulative EDX-mappings of the respective micrographs depicted in Fig. 2a, whereas Fig. 2c and d show the separate Ga- and Ag-mappings, respectively. As the first step of the lamella-extraction, three Pt-protection layers are deposited onto ROI 2 illustrated in Fig. 1c, which can be seen in Fig. 2a and b. The locations of the Pt-depositions, lamella cross-sections and trenches in ROI 2 are shown in Supplementary Fig. 1. Details about Pt-deposition, FIB-milling and temperature control thereby are given in Methods and Supplementary Note 1. The EDX-mappings of Ga in Fig. 2c indicate Ga-enrichment on the sample cross-section in the vicinity of the trenches. However, the intensity of the Ga-signal decreases with decreasing milling temperature. Moreover, a comparison between the Ga- and Ag-mappings of the trenches milled at 25 °C and −30 °C reveals on the sample cross-section an increased Ga-concentrations at the locations of Ag_3_Sn precipitates, see Fig. 2c and d, respectively..

The trench and lamella prepared at 25°C differ from the -30 and -60 °C ones in several ways. As can be seen in Fig. 2b and d, the Ag_3_Sn precipitates in the lamella cross-section of the 25 °C-trench appear to be qualitatively larger than the ones in the −30 and −60 °C lamellae, respectively. Note, that the micrographs in Fig. 2 are imaged under a stage tilt of 56°, so any quantitative analysis of the precipitate size is not feasible. Nevertheless, we argue that a qualitative comparison of the particle sizes can be done, since the cutting of all three trenches and EDX-mapping are done under the same stage tilt. The comparative analysis in Supplementary Fig. 2 shows that at least one Ag_3_Sn precipitate in the 25 °C-lamella cross-section seems to have coarsened during milling. Further we evaluate the area of the precipitates. The area of the largest precipitate in the 25 °C-lamella cross-section is 324% and 257% larger than the largest precipitates in the -30 and -60 °C-lamella cross-sections, respectively. The mean area of Ag_3_Sn-precipitates in the 25 °C-lamella cross-section is also 179% and 171% larger than their mean area in the -30 and -60 °C-lamella cross-sections. The comparison of the areas of Ag_3_Sn precipitates in the trenches milled at 25, -30 and -60 °C is plotted in Supplementary Fig. 2a. The analysis is based on the Ag-L EDX-mappings shown in Supplementary Fig. 2b and their binary thresholds is shown in Supplementary Fig. 2c**.** The precipitate area analysis is described in Methods and Supplementary Note 2. In addition, the SE-detail of the 25 °C-trench, see Fig. 2a, shows a bright line in the lamella cross-section, which is formed during trench-milling at 25 °C. The detailed Ga-mapping in Fig. 2c reveals Ga-enrichment within this bright line. Lastly, the backside of the upper 25 °C-trench exhibits an irregular structure, in which finely dispersed Ga- and Ag-rich needles have grown in the redeposition. A detailed FESEM-SE micrograph, as well as EDX-mappings of these Ga- and Ag-enriched needles are shown in Supplementary Fig. 3a–d.

### Impact of stage temperature on APT-specimen milling behaviour and Ga-implantation utilising a Pt-protection layer

**Fig. 3:**
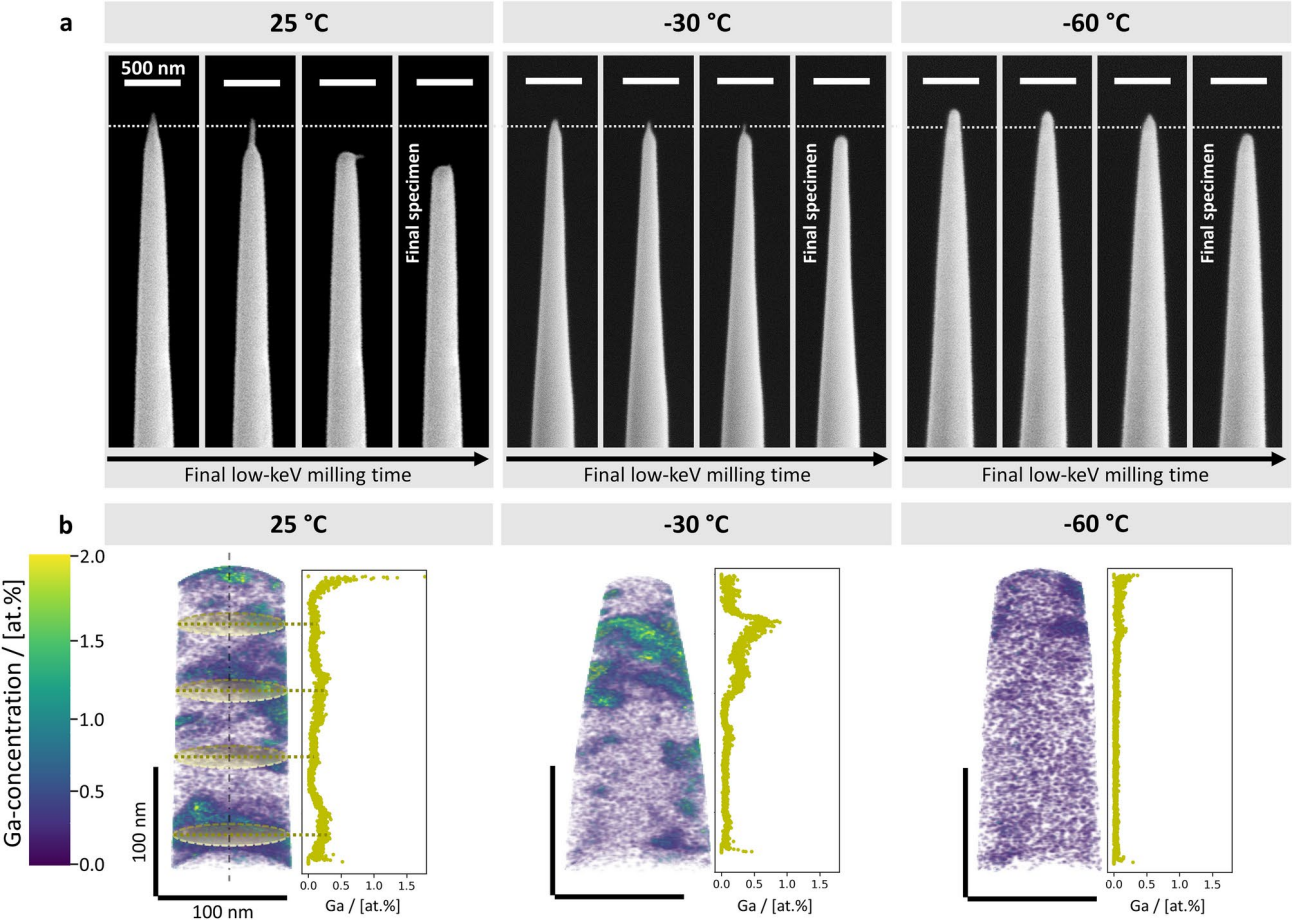
FESEM in-operando observation of the evolution of APT-specimen geometry during final low-keV milling at the investigated temperatures and Ga-concentration in the resulting APT-specimens utilising a Pt-protection layer. **a** FESEM-SE screen-grabs from during the final low-keV APT-specimen milling step utilising 5 keV ion accelerating voltage and 16 pA FIB current. The dashed lines mark the respective Pt–Sn interfaces. The specimen evolution during the final milling step is shown at 25 °C, -30 °C and -60 °C for four timesteps. The respectively last panels show the final APT-specimens for each milling temperature. Scale bar of 500 nm is valid for all images. **b** 3D Ga-concentration distributions from the APT reconstructions for specimens prepared at 25 °C, -30 °C and -60 °C, alongside the respective Ga-concentration profiles along the direction of evaporation. The correspondence of datapoints in the Ga-concentration profiles with the Ga-concentration distributions is exemplarily sketched for the 25 °C-specimen. Scale bars of 100 nm are valid for all images. The Ga-concentration distributions are scaled from 0.0 to 2.0 at.% Ga.

In order to prepare APT-specimens from the lamellae shown in Fig. 2, the lamellae need to be extracted from the bulk and fixed to the posts of specially dedicated microtip arrays. The procedure for lamella extraction and fixation to the posts is described in detail in Methods. Subsequently, the rough annular milling, described in Methods and schematically depicted in elevation-view in Supplementary Fig. 4a and in top-view Supplementary Fig. 4b, is performed at 25, -30 and -60 °C, respectively. After undergoing increasingly finer annular milling steps, the final low-keV milling step is applied in order to remove the remaining Pt-protection layer and to bring the specimen into its final shape. In operando SE-imaging during milling allows the real-time observation of the evolution of the specimen’s shape, which is shown in Fig. 3a for the respective milling temperatures. Screen-grabs from additional milling timesteps are shown in Supplementary Fig. 5 for a more complete visualisation of the specimen milling behaviour at the investigated temperatures. As can be seen, the stage temperature has a substantial impact on the milling behaviour of the specimen during this final milling step. This final low-keV milling step is also schematically depicted in Supplementary Fig. 4a and b, described in Methods, as well as in Supplementary Note 3.

During the low-keV milling at 25 °C, shown in Fig. 3a, the Ga-ion beam preferably mills the β-Sn beneath the residual Pt-protection layer, instead of gradually removing the material uniformly. Thereby, the region, which is located directly under the Pt-layer, is removed before the Pt. As a result, the Pt-residue tips over and remains stuck to the specimen. A similar milling behaviour, although less pronounced, is observed during low-keV milling at -30 °C, as can be seen in Fig. 3a. However, when the milling is performed at -60 °C, as shown in Fig. 3a, the non-uniform milling behaviour is inhibited and the APT-specimen retains a stable shape during the entire preparation process, including the final Pt-removal. .

For the investigation of the milling temperature dependence of Ga-implantation into the low-melting SAC305 solder alloy, the prepared specimens are measured utilising APT. The measurement parameters for APT are given in Methods. Figure 3b depicts the 3D Ga-concentration distributions in one set of APT-specimens prepared at 25, -30 and -60 °C, respectively. Additionally, the Ga-concentration profiles along the direction of evaporation, i.e. the rotation axes of the specimens, for the respective specimens are also shown. The correspondence of the datapoints in the Ga-concentration profiles with the respective 3D Ga-concentration distributions is indicated exemplarily for the 25 °C-specimen in Fig. 3b. A second specimen set for each milling temperature is shown in Supplementary Fig. 6. The depiction of the two APT-specimen sets rotated for 0, 90, 180 and 270°, respectively, around the axis of evaporation, are shown in Supplementary Fig. 7, in order to fully present the 3D Ga-distribution within the specimens. For a more complete picture of the APT-measurements, exemplary mass spectra and detector hit maps are shown in Supplementary Fig. 8a and b, for specimens milled at 25, -30 and -60 °C. Moreover, the bulk Ga-concentrations in the APT-specimens are evaluated for both sets and given in Table 1, as well as the mean value for the two sets. The Ga-concentration profiles in Fig. 3b and Supplementary Fig. 6 show a decreasing Ga-implantation depth with decreasing milling temperature. Moreover, the Ga-enrichment is not homogeneous over the specimens and indicates agglomerations, as can be seen in the Ga-concentration distributions in Fig. 3b and Supplementary Fig. 6. However, the agglomeration formation becomes less pronounced as the stage temperature during milling is decreased.

**Table 1:** Bulk Ga-concentrations for two sets of APT-specimens prepared at 25, -30 and -60 °C shown in Fig. 3b (set 1) and Supplementary Fig. 6 (set 2).

**Bulk Ga-concentration/[at.%]**			
**Set**	**25 °C**	**−30 °C**	**−60 °C**
1	0.098	0.089	0.016
2	0.091	0.079	0.039
Mean	0.095	0.084	0.028

Note, that not only is the Ga-implantation depth decreased by lowering the milling temperature, but also the overall extent of the implantation. This is shown in Table 1, where the bulk compositions of Ga in the respective APT-specimens are evaluated. The mean Ga-concentration decreases from 0.095 at.% in the specimens prepared at 25 °C to 0.084 at.% in the ones prepared at -30 °C. Furthermore, there is a significant decrease to 0.028 at.% in mean bulk Ga-concentration in the specimens that are milled at -60 °C.

### Impact of an Al-protection layer on the APT-specimen radius and Ga-implantation during milling at −60 °C

**Fig. 4:**
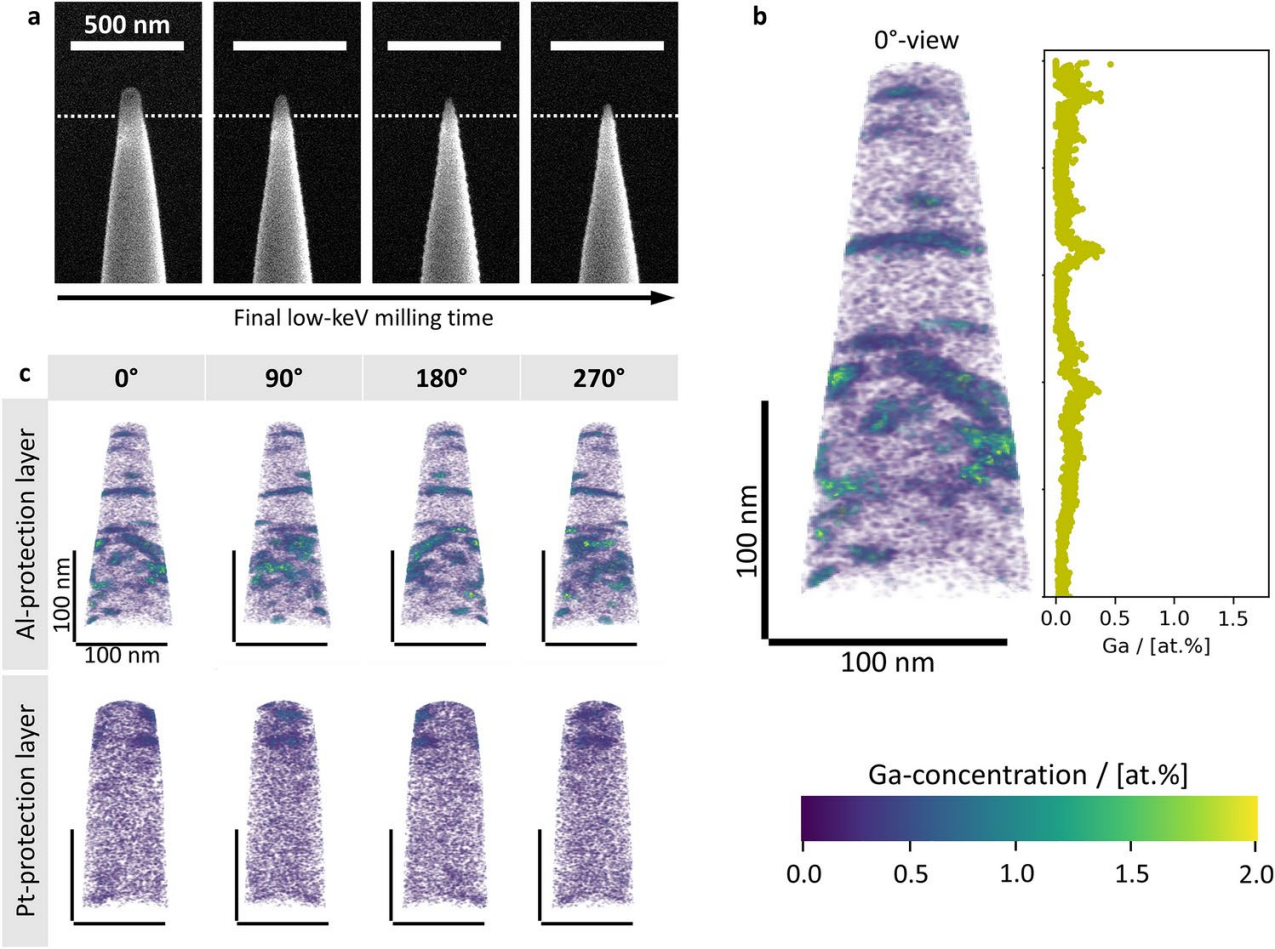
Evolution of APT-specimen shape during final low-keV milling utilising an Al-protection layer, APT reconstruction and analysis, as well as comparison of Ga-concentration distributions in specimens milled at −60 °C with Al- and Pt-protection layers, respectively. **a** FESEM-SE screen-grabs from the final low-keV milling of the APT-specimen with the Al- protection layer. The Al–Sn-interface is marked with a dashed line. Scale bar of 500 nm is valid for all images. The final specimen still has a small amount of Al on top, which is evaporated during the APT run-up. **b** 3D Ga-concentration distribution of the APT-specimen in **a** and its Ga-concentration profile along the direction of evaporation. The Ga-concentration distribution is scaled from 0.0 to 2.0 at.% Ga. The scale bars are given for 100 nm. **c** Comparison of 3D Ga-concentration distributions in specimens milled at −60 °C utilising Al- and Pt-protection layers. The 3D Ga-concentration distributions are shown for a rotation of 0, 90, 180 and 270° around the direction of evaporation, respectively. Scale bars of 100 nm are valid for all images. All Ga-concentration distributions are scaled from 0.0 to 2.0 at.% Ga.

Despite exhibiting stable milling behaviour, the final tip-radius of the APT-specimens milled at −60 °C is rather blunt, as is exemplarily shown in Fig. 3a, wherein the final specimen radius results in approximately 65 nm. The blunting of the specimen occurs after the Pt-protection layer is removed and the underlying Sn is directly exposed to the Ga-ion beam, whereas the Pt-layer itself can be sharpened. This can be seen in the third panel of the -60 °C specimen in Fig. 3a where there is still some residue of the Pt-layer left on the specimen. In an effort to obtain smaller initial tip radii of APT-specimens, we substitute Pt for an Al-protection layer which is deposited via physical vapor deposition (PVD). The PVD-parameters are given in Methods. Screengrabs from the corresponding low-keV specimen-milling are illustrated in Fig. 4a. Due to the lower evaporation field of Al^+^(19 V/nm^42^) compared to Sn^++^(23 V/nm^42^), it does not have to be fully removed during the final low-keV milling step and can be evaporated during the APT run-up. The final specimen shape, shown in the fourth panel in Fig. 4a, exhibits a specimen radius of approximately 38 nm. Hence, is significantly sharper than the one shown in Fig. 3a for the Pt-protection layer. The Al–Sn-interface is marked with a dashed line.It can be seen that a small amount of the Al-layer remains on the specimen. The Al-residue is evaporated during the run-up of the APT-measurement and does therefore not influence the measurement for the underlying region. The 3D Ga-concentration distribution, as well as the Ga-concentration profile along the axis of evaporation are shown in Fig. 4b. A second specimen for comparison is shown in Supplementary Fig. 6 and 7.

Figure 4c shows a comparison between the specimens milled at −60 °C utilising an Al- and Pt-protection layer, respectively. Here, the 3D Ga-concentration distributions are rotated for 0, 90, 180 and 270° around their axes. Although the specimen preparation at −60 °C utilising an Al-protection layer yields a smaller specimen radius, the extent of Ga-implantation is not lowered, compared to the specimen with an Pt-protection layer milled at the same temperature. Indeed, the mean bulk Ga-concentration of the former is significantly higher with 0.087 at.%, compared to the mean value of the latter with 0.028 at.%, see Table 1. Furthermore, the Ga-distribution in the former is more inhomogeneous than in the latter and the Ga-implantation depth is deeper in the former, as can be seen when comparing the APT**-**measurements in Fig. 4c.

## Discussion

APT is a method for the analysis of the elemental composition on the near-atomic scale^9,40^. Due to its elemental and spatial resolution, it is a useful tool to analyse elemental distributions on a very small scale, e.g. elemental segregation to lattice defects. In order to study such segregation effects in the low-melting SAC305 solder alloys, the preparation possibilities of APT-specimens utilising Ga-FIB needs to be evaluated. Since the eutectic temperature of SAC305 with 217 °C^43^ is rather low, the local temperature rise during FIB-milling may significantly impact its milling behaviour and microstructural features. These effects are investigated in this study. In the following, the theoretical local temperature rises that are caused by the respective Ga-FIB milling parameters are approximated and compared to our experimental findings. Moreover, the extent of Ga-implantation during the preparation process is discussed, as well as an optimised preparation for the minimisation of the said Ga-implantation is illustrated. The study of grain boundary segregation in SAC305-Bi solder alloys will be the subject of our future study.

When milling samples utilising Ga-FIB, the impact of Ga-ions into the sample surface and the conversion of their kinetic energy into heat causes a local temperature rise. The extent of this temperature rise is dependent on multiple factors, such as ion energy, ion beam diameter, sample thermal conductivity and sample geometry^13^. Some work has been previously done to study the effects of such FIB-induced heating artefacts. Shukla et al^12^. directly studied the temperature rise due to Ga-ion beam irradiation on a thermally isolated SiO_2 _sample using a nanothermo-probe, which was fabricated by FIB-deposition of Pt and tungsten. They measured a local temperature rise of 250 °C when the Ga-FIB impinged on the sample with 30 keV and 10 pA. Moreover, Cen and van Benthem^11^ observed the heating effects of a Ga-FIB indirectly by correlating the microstructural changes in gold/nickel bilayer films during Ga-ion beam exposure with in-situ TEM heating experiments. They concluded that the microstructural changes during ion beam exposure was consistent with those during annealing at 400 °C for several minutes. It should be noted that in both studies, the samples were thermally isolated from the sample holder.

Ishitani and Kaga^13^ provided an estimate of the local temperature rise $\Delta \text{T}$ / [$\text{K}$] in a bulk sample during ion beam bombardment by

1 $$\Delta \text{T}=\frac{\text{V}\cdot \text{I}}{\text{d}\cdot\kappa \cdot \sqrt{\pi }}$$

where $\text{V}$ / [$\text{keV}$] is the ion accelerating voltage, $\text{I}$ / [$\text{A}$] the FIB current, $\text{d}$ / [$\text{m}$] the beam diameter and $\upkappa$ / [$\text{W}/(\text{m} \text{K})$] the thermal conductivity. Moreover, Ishitani and Kaga^13^ calculated the shape-factor for cross-sectioned samples as ${\text{R}}_{\text{cross}-\text{section}}=2$. Based on this, when polishing the lamellae cross-sections with V = 30 keV, I = 1 nA, d = 10 nm^13^, and inserting the thermal conductivity for β-Sn κ_β-Sn_= 66.6 W/(m∙K)^44^ into **Eq. **1, the calculated local temperature rise yields ∆T = 51 °C. Considering that it takes roughly 30 min to mill the trenches, this approximation may explain the mean Ag_3_Sn-precipitate coarsening of 179% and 171% that is observed in the 25°C-lamella cross-section compared to the lamellae that are milled at −30 °C and −60 °C, respectively, as shown in Supplementary Fig. 2a. Due to the image distortion on the lamellae cross-sections in Fig. 2 and Supplementary Fig. 2b, the exact sizes of the Ag_3_Sn-precipitates cannot be evaluated. Therefore, the precipitate coarsening is only given comparatively. Equation 1 does not take the sputtered material into account. Yet, the equation by Ishitani and Kaga^13 ^provides a qualitative estimate regarding the temperature trend. The estimated temperature rise might be potentially significantly higher than the calculated 51°C. This argument is supported by Supplementary Fig. 3. Partial melting and re-solidification, considering the sputtered material, cannot be neglected as possible reason for the shown redeposition artefacts. The re-solidified material may grow as a finely dispersed mixture of Ga and Ag, as Ag has a solubility limit of about 8 at.% Ga at room temperature^45^. Due to rapid solidification during the milling process, the needle-shaped redeposition artefacts may, however, be over-saturated in Ga, as shown in Supplementary Fig. 3.

Ishitani and Kaga^13^ further calculated the shape-factor for pillar-shaped samples as ${\text{R}}_{\text{pillar}}=\frac{4\text{L}}{\sqrt{\uppi }\cdot \text{d}}+1$, where $\text{L}$ is the height of the pillar and it is assumed that the diameter of the pillar is equal to $\text{d}$. Assuming an APT-specimen length of L = 5 µm, V = 5 keV, I = 16 pA, d = 10 nm^13^ and κ_β-Sn_= 66.6 W/(m∙K)^44^, the local temperature rise during the final low-keV milling step is calculated as ∆T = 76.5 °C. This approximation is valid for pillars whose diameter equals the ion beam diameter $\text{d}$, and is therefore an over-estimation. Nonetheless, the local temperature rise in the APT specimens is considered to be non-negligible, especially because the thermal conductivity of the specimens may be significantly impaired by their Pt-fixations onto the specimen-holder posts, since the deposited Pt consists largely of carbon^46,47^. The effect of this temperature rise during the final low-keV milling step can be seen clearly for the specimen milled at 25 °C in Fig. 3a, where the β-Sn that is located directly beneath the Pt-layer is milled more intensely than the Pt-layer itself. This is an indication for the thermal instability of β-Sn during the milling process, since its stability during milling is gradually improved by lowering the milling temperature to -30 °C and -60 °C, as is also shown in Fig. 3a.

Ga-ions impinging onto the sample not only cause the local temperature rise, but may also lead to the implantation of Ga into the material^48,49^. This Ga-implantation may significantly alter structural properties of the investigated material, e.g. grain boundaries^50^. A Ga-implantation artefact is shown in the detailed FESEM-SE micrograph and Ga-EDX mapping in ROI 3 for the 25 °C-lamella in Fig. 2c. The EBSD KAM-map of ROI 1 in Fig. 1b, as well as the channelling contrast in the FESEM-BSE micrographs in Fig. 1c and Supplementary Fig. 1a show slightly misoriented areas. Hence, this Ga-implantation artefact may be formed at small-angle grain boundaries that extend beneath the imaged ROI 2. Moreover, the Ga- and Ag-EDX mappings in Fig. 2c and d show an enhanced Ga-concentration at the locations of Ag_3_Sn-precipitates, compared to the β-Sn matrix. Therefore, to avoid such artefacts, Ga-implantation during specimen preparation should be minimised.

In addition to the EDX-analysis of the trenches, Ga-implantation in the β-Sn matrix during FIB-milling at 25, −30 and −60 °C is investigated utilising APT. The 3D Ga-concentration distributions in Fig. 3b and Supplementary Fig. 6, as well as the analysis of its bulk concentration in the APT-specimens in Table 1 show the decrease in Ga-implantation with decreasing milling temperature. Furthermore, the decrease in Ga-implantation depth with decreasing milling, seen in Fig. 3b and Supplementary Fig. 6, may be due to decreased Ga-mobility as the sample temperature is lowered. The Ga-ions may therefore be limited in their diffusion further into the sample. Conversely, insufficient cooling during milling may enhance the mobility of Ga-ions and lead to more pronounced agglomeration. Moreover, the quantitative analysis of the extent of Ga-implantation during FIB-milling in Table 1 shows a significant decrease in bulk Ga-concentration in the specimens milled at −60 °C, compared to the ones milled at 25 and −30 °C, respectively. This may be attributed to the enhanced stability of the material during FIB-milling at −60 °C that is observed in Fig. 3a.

In order to fabricate APT-specimens with smaller initial radii at -60 °C, Pt is substituted with Al, see Figs. 3 and 4 for comparison. Although the utilization of Al results in a sharper APT-specimen, see Fig. 4a, the Ga-implantation is not further reduced. Conversely, the mean bulk Ga-concentration in the specimens with the Al-protection layer amounts to 0.087 at.%, which is a significant increase compared to the mean bulk Ga-concentration of 0.028 at.% in the specimens with a Pt-protection layer, see Table 1. Despite the larger initial specimen radii of the specimens with the Pt-protection layer, their evaporation behaviour during APT provides a sufficient ion detection yield, as all measurements are manually stopped after 36 million ions are detected, see Methods. This beneficial evaporation behaviour, which allows the analysis of relatively large specimen volumes, may be attributed to the low evaporation field of 23 V/nm of Sn^++^^42^. Therefore, it is concluded that the advantage of an Al-protection layer is limited to the visual appearance of the APT-specimens and that it does not significantly benefit the preparation or the measurement, but rather increases the preparation effort.

In summary, this study elaborates the effects of stage temperature on the Ga-FIB preparation of APT-specimens from the low-melting SAC305 solder alloy, as well as the impact of two different protection layers. It is concluded that lowering the milling temperature to −60 °C for all Ga-FIB – sample interactions enables the effective preparation of APT-specimens, while also minimising Ga-implantation during the milling process. Further lowering the milling temperature may yield even better results, however, this study is limited to stage temperature control utilising a Peltier cooling system. Similarly, this study is limited to the effects of Ga-FIB on the material. Specimen preparation with a plasma-FIB may yield different results^49^. Nonetheless, we conclude that the utilisation of a Ga-FIB, a Pt-protection layer and Peltier cooling stage enables the effective and reproducible preparation of APT-specimens from low-melting alloys, such as SAC305. Hence, the preparation workflow for APT in this study opens the door for previously unexplored possibilities to investigate elemental segregation in low-melting solder alloys.

## Methods

### Materials and sample production

The investigated sample is made from the Sn – 3.0 wt.% Ag – 0.5 wt.% Cu (SAC305) solder alloy, which is reflowed at a peak temperature of 240 °C with a mean heating rate of 44 °C/min in an inert N_2_ atmosphere, followed by rapid air cooling to 90 °C with a mean cooling rate of 107 °C/min and finally by ambient air cooling to room temperature. Subsequently, the sample is cycled for 300 TCoB cycles between −40 and 125 °C in ambient atmosphere. The ramp- and dwell-times are set to 15 min, respectively.

### Sample preparation and FESEM imaging

In order to ensure thermal contact with the sample stage, the curvature of the investigated solder ball is levelled utilising a 3D Micromac microPREP PRO femtosecond laser with a laser power of 300 mW. The same laser parameters are used to pre-prepare the cross-section for FESEM. The final cross-section for FESEM imaging, EBSD mapping and lamella lift-outs are prepared with a Hitachi IM4000 + ion milling system. The accelerating voltage for ion slicing is set to 6 kV and the swing angle to 30°. The overview EBSD map is acquired at room temperature with a Zeiss 450 Gemini FESEM. An accelerating voltage of 10 kV, a step size of 400 nm and an Oxford Symmetry detector is used for EBSD mapping. Oxford Instrument AZtecCrystal 6.1 is utilised for the evaluation of the EBSD data. EDX mappings are done at room temperature with a FEI Versa 3D HIVAC FESEM and an EDAX Octane Elect EDX detector. The acceleration voltage is set to 10 kV.

### FIB-preparation and stage temperature control

The preparation of the APT tips is done with a FEI Versa 3D HIVAC Ga-FIB. To control the stage temperature during preparation, a Kleindiek Peltier micro heating and cooling stage is utilised. The stage temperature is systematically varied for all FIB – sample interactions. Three stage temperatures are studied: 25, −30 and −60 °C.

The first step of the APT-specimen preparation involves the deposition of the Pt-protection layer onto the ROI of the future lamella. In this study, the Pt-deposition is conducted with the electron beam at 2 keV and 1 nA, as not to damage the sample with the FIB. All Pt-depositions are done at room temperature, since the Pt-precursor in the gas injection system (GIS) condenses at lower temperatures. After the Pt-protection layers are deposited, the trenches for the lamella lift-outs are cut at the respective stage temperatures. The rough trench cuts are done with 30 kV and 1 nA FIB-current, followed by cross-section polishing with 0.5 nA and ± 4° stage over- and under-tilt, respectively, to minimise the lamella-tapers. Subsequently, the lamellae are cut free at the respective temperatures, using the FIB, and the lift-outs are fixed to the manipulator-needle with redeposition-welds, as described in^51,52^. The lamella is then split up onto the posts of a commercial microtip coupon, which is held at the respective stage temperatures of 25, −30 and −60 °C. The −30 and −60 °C specimens are preliminarily fixed to the posts with redeposition-welds, milled with 30 keV and 50 pA. Then the stage is turned 180° and the gap between sample and post is filled with Pt, deposited with the electron beam using 2 keV and ∼1 nA at RT in order to ensure a safe fixture for the subsequent specimen preparation and the APT measurement. The 25 °C specimens are directly fixed with electron beam deposition onto the posts, utilising 2 keV and ∼1 nA. In order to bring the specimens into the final needle-shape that is needed for the field evaporation during APT, annular milling of the specimens is performed at the respective temperature, with 30 kV and decreasing FIB-currents, from 500 to 30 pA and decreasing annular diameters. The outer annular diameters decrease from ∼5 µm to ∼200 nm, while the inner diameters decrease from ∼2.5 µm to ∼130 nm. The final milling step, to remove the Pt-layer and sharpen the specimens, is done with 5 kV and 16 pA at the respective stage temperatures. The process of milling the specimens is described in Supplementary Note 3 and shown schematically in Supplementary Fig. 4.

### Ag_3_Sn-precipitate area analysis

In order to qualitatively compare the Ag_3_Sn-precipitate sizes in the lamellae cross-sections that have been milled at 25, −30 and −60 °C, the respective ROIs are cropped from the EDX Ag-mappings in Fig. 2d. These ROIs are then binarily thresholded utilising Python 3.8.13 and OpenCV 4.0.1 in order to distinguish between precipitates and background. The areas of these thresholds are then binned according to their size and plotted against the count of each size in the respective image. Moreover, their maximum and mean area sizes are calculated. The results from the precipitate area analysis are shown in Supplementary Fig. 2.

### APT measurement and analysis

In order to compare the extent of Ga-implantation in the samples prepared at various stage temperatures, APT measurements are done utilising a Cameca LEAP 5000 XR. All specimens are evaporated via voltage pulsing. The specimen temperature is set to 50 K. The voltage pulse fraction is set to 15%, the pulse frequency to 125 kHz and the detection rate to 1%. All measurements are stopped once the target ion detection count of 36 million is reached in order to obtain comparable reconstruction volumes.

The reconstruction and elemental analysis of the APT tips is done with the Cameca IVAS 6 package embedded within AP Suite 6.3 software. All reconstructions are done based on the respective voltage curves. For the 3D Ga-concentration distributions, 0.02 at.% Ga iso-concentration surfaces are created and the Ga-concentration within them is visualised as 3D distributions. The elemental bulk compositions and concentration profiles are analysed for atomic and decomposed ions.The Ga-concentration profiles are plotted along the direction of evaporation. The term “mean bulk concentration” in the manuscript refers to the mean value of the bulk concentrations in the specimens from the sets 1 and 2 for each milling temperature.

### Deposition of the Al-protection layer

Al is deposited onto the cross-section of the sample as a substitution for the Pt-protection layer utilising PVD at RT. The sample surface is first sputtered for 2 min, utilising 316 V, 0.35 A and 113 W. Subsequently, Al is deposited with 338 V, 0.35 A and 121 W. Both processes are conducted at a chamber pressure of 3∙10^–3^ mbar and an argon gas-flow of 30 Sccm at RT.

## Supplementary Information

Supplementary Information.

## Data Availability

Data is provided within the manuscript or supplementary information files or available from the corresponding author upon reasonable request.

## Code availability

All code that support the findings of this study are available from the corresponding author upon reasonable request. Data is provided within the manuscript or supplementary information files or available from the corresponding author upon reasonable request.
