# Supplementary material for: Effective preparation of low-melting solder materials for atom probe tomography
Source: Sci Rep. 2024 Nov 27;14:29475. doi: 10.1038/s41598-024-79753-w (PMC11603297; doi:10.1038/s41598-024-79753-w)
Supplement: Supplementary file 1 — Supplementary Information. [file 41598_2024_79753_MOESM1_ESM.docx]

# **Effective preparation of low‑melting solder materials for atom probe tomography**

Charlotte Cui^1^, Michael Tkadletz^2^, Michael Reisinger^3^, Peter Imrich^3^, Walter Hartner^4^, Roland Brunner^1*^

*Corresponding author: [roland.brunner@mcl.at](mailto:roland.brunner@mcl.at)

^1^ Materials Center Leoben Forschung GmbH, Department Microelectronics, Roseggerstraße 12, 8700 Leoben, Austria

^2^ Montanuniversität Leoben, Chair of Functional Materials at the Department Materials Science, Roseggerstraße 12, 8700 Leoben, Austria

^3^ Kompetenzzentrum für Automobil‑ und Industrieelektronik GmbH, Europastraße 8, 9524 Villach, Austria

^4^ Infineon Technologies AG, Wernerwerkstraße 2, 93049 Regensburg, Germany

In this study, the effects of stage temperature during specimen preparation from low‑melting Sn – 3.0 wt.% Ag – 0.5 wt.% Cu (SAC305) for atom probe tomography (APT) utilising a gallium (Ga‑) focussed ion beam (FIB) is studied utilising field‑emission scanning electron microscopy (SEM), energy dispersive X‑ray (EDX) and APT. Supplementary information is given in the following.

### **Supplementary Note 1: Deposition of Pt-protection layers and milling trenches in ROI.**

As the first step of the lift-out preparation, three Pt-protection layers are deposited on the ROI depicted in **Fig. 1b** utilising the GIS and the SEM electron beam. All protection layers are deposited with an acceleration voltage of 2 keV and sample current of 1 nA at room temperature in order to avoid condensation of the Pt-precursor on the sample surface. The resulting Pt-protection layers in the ROI are shown in **Supplementary Fig. 1a**.

Subsequently, the trenches adjacent to the Pt-protection layers are milled utilising a Ga-FIB with 30 keV and 1 nA at a stage-tilt of 52°. Additionally, polishing of the lamellae is done with 30 keV and 0.5 nA by over- and under-tilting by ±4°. For both trench milling and lamella polishing, the Peltier-stage temperature is set to 25, -30 and -60°C, respectively. An SE micrograph of the resulting trenches in the ROI is shown in **Supplementary Fig. 1b**, alongside the Ga-EDX mapping of the ROI in **Supplementary Fig. 1c**. Higher magnification SE-micrographs and EDX mappings of the trenches and cross-sections are shown in **Fig. 2**.

### **Supplementary Figure 1: Trenches cut in ROI.**


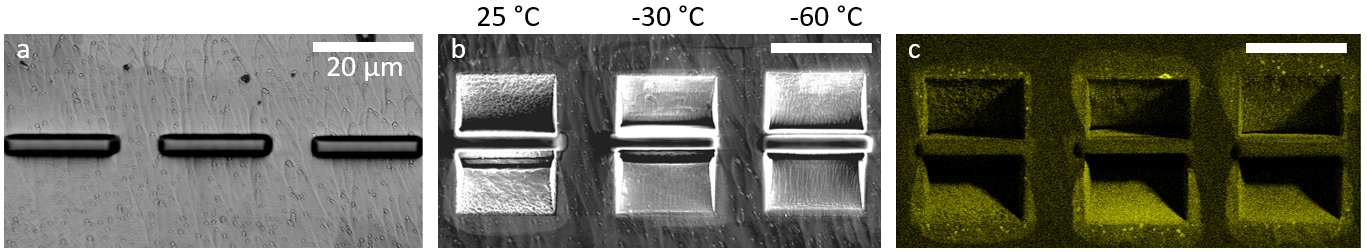


**a** Backscattered electron (BSE) micrograph of Pt‑protection layers deposited on the ROI. **b** Trenches cut at 25, ‑30 and ‑60°C, respectively. **c** Ga EDX‑mapping of the ROI after the trenches are cut at the respective temperatures.

### **Supplementary Note 2: Analysis of precipitate size distributions in lamella cross-sections that are cut at 25, -30 and -60 °C, respectively.**

In order to quantitatively compare the Ag_3_Sn precipitate sizes in the lamellae that are prepared at 25, -30 and -60°C, the EDX Ag-mappings of the lamellae shown in **Fig. 2** are further processed. Firstly, the mappings are cropped to only show the lamella cross-sections and not the sample surface. Therein, all three mappings are cropped to the same image size as to depict a comparable area. These cropped mappings are then smoothed, normalized and binarily thresholded in order to segment the Ag_3_Sn precipitates. This conventional image processing is done utilising Python 3.8.13 and OpenCV 4.0.1. The same OpenCV package is then used to calculate the areas of the respective thresholded precipitates in the images. These precipitate areas are then categorised into discrete classes with respect to their size and plotted against the number of precipitates of each size class for each milling temperature. Because mapping the trenches under 52° with EDX yields distorted images, we forgo the conversion of pixels into metric units and compare the segmented precipitate-areas in pixels. Nonetheless, since all trenches are milled and imaged under the same stage tilt and all EDX-mappings are acquired with the same parameters, this quantitative analysis yields comparable results. The precipitate size distributions, cropped EDX Ag-mappings and thresholded images are shown in **Supplementary Fig. 3a – c**.

### **Supplementary Figure 2: Precipitate size distributions in the lamella cross‑sections that are cut at 25, ‑30 and ‑60°C, respectively.**


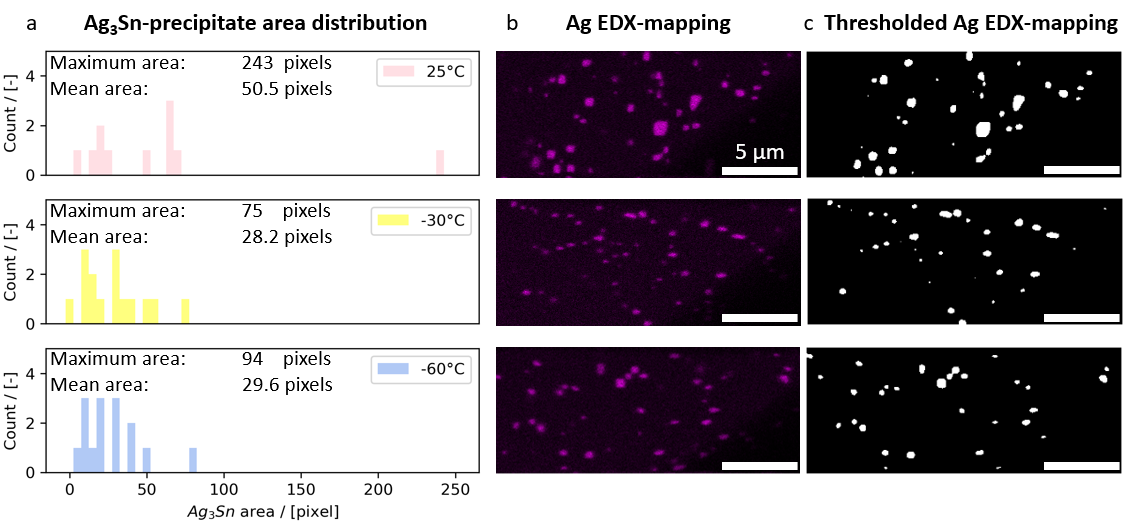


**a** Ag_3_Sn precipitate area distributions based on the Ag EDX‑mappings of the lamella cross‑sections under 56° stage‑tilt in shown in **b**, which are cropped from **Fig. 2d**. The precipitate areas are analysed based on the binary thresholds in **c**. Scale bar of 5 µm is valid for all images.

### **Supplementary Figure 3: Redeposition in 25 °C‑trenches.**


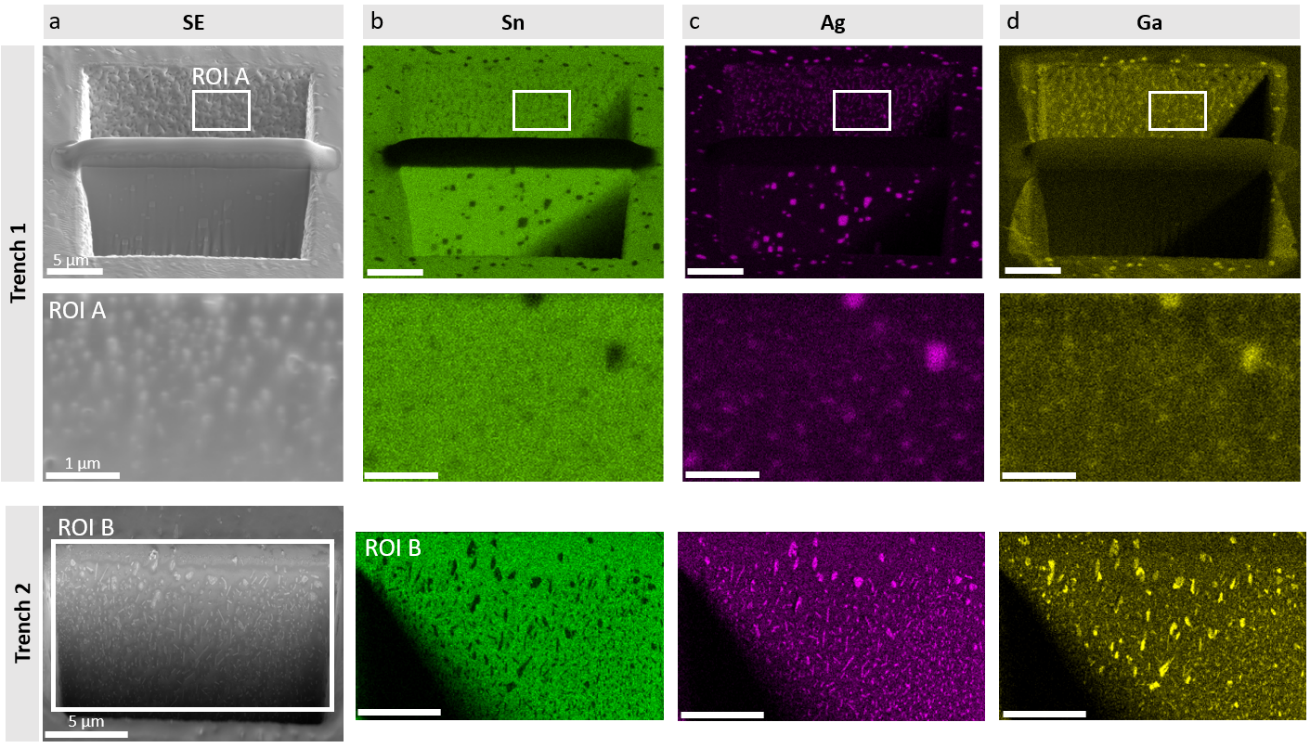


EDX‑mappings of the 25 °C‑trench (Trench 1) and mappings of the redeposition from milling in ROI 3 which is marked with rectangles. A second trench (Trench 2) for the visualisation of the needle‑shaped redeposition artefacts is shown below. **a** FESEM SE micrographs, **b** Sn‑mapping (green), **c** Ag‑mapping (magenta) and **d** Ga (yellow). Scale bar of 5 µm is valid for the first row of images, scale bar of 1 µm is valid for the second row and scale bar of 5 µm is valid for the third row.

### **Supplementary Note 3: APT-specimen annular milling procedure.**

The annular milling procedure has been described in previous studies^1–5^. For the preparation of Sn specimens, comparatively low FIB-currents suffice for a high sputter yield. In this study, the first annular milling step for bringing the specimen in a rough conical shape, schematically shown in **Supplementary Fig. 4**, is done with 30 keV and 500 pA. Subsequent annular milling steps are done with decreasing FIB-currents and decreasing outer and inner annular diameters. In this work, we gradually move from 300 to 30 pA. The final 5 keV milling step is done with 16 pA by rastering over the specimen. This final milling step removes the protection layer and gives the APT-specimen its final shape. The annular milling workflow for the preparation of APT-specimens is schematically shown in **Supplementary Fig. 4**. This milling procedure is done at 25, -30 and -60°C, respectively.

### **Supplementary Figure 4: Schematic APT‑specimen annular milling procedure.**


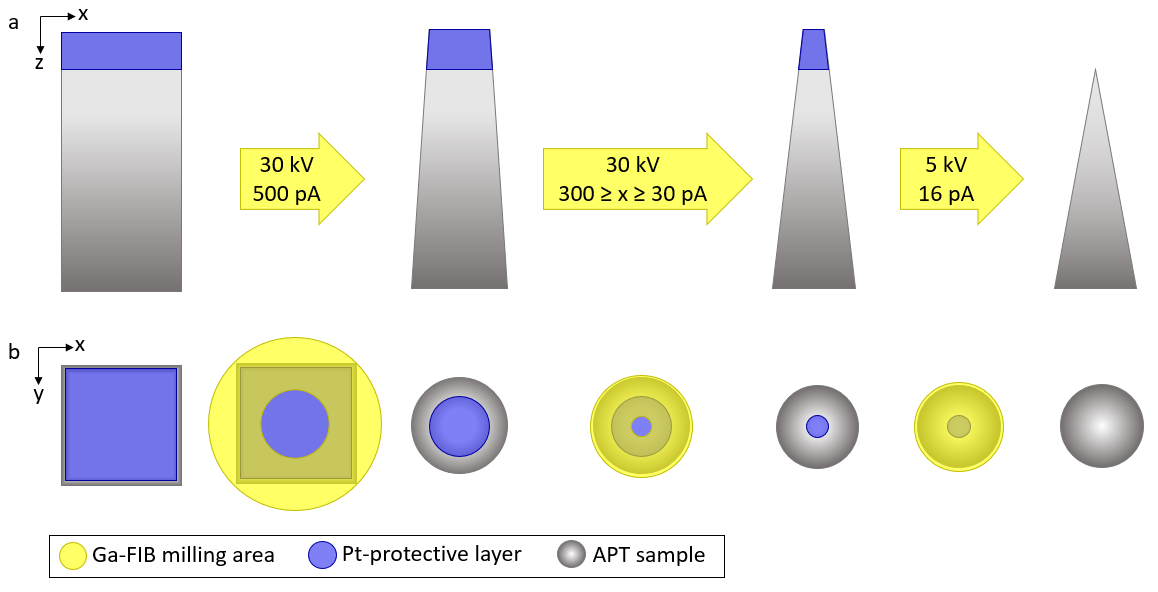


**a** Schematic elevation‑view of the tip‑geometry evolution during the various annular milling steps, including the Ga‑FIB parameters for each step. The respective top‑views are shown in **b**, alongside the Ga‑FIB milling areas, depicted in yellow. The APT‑specimen is shown in grey and the Pt‑protection layer in blue.

**Supplementary Figure 5: FESEM in situ observation of the evolution of APT‑specimen geometry during final low‑keV milling at the investigated temperatures utilising a Pt‑protection layer.**


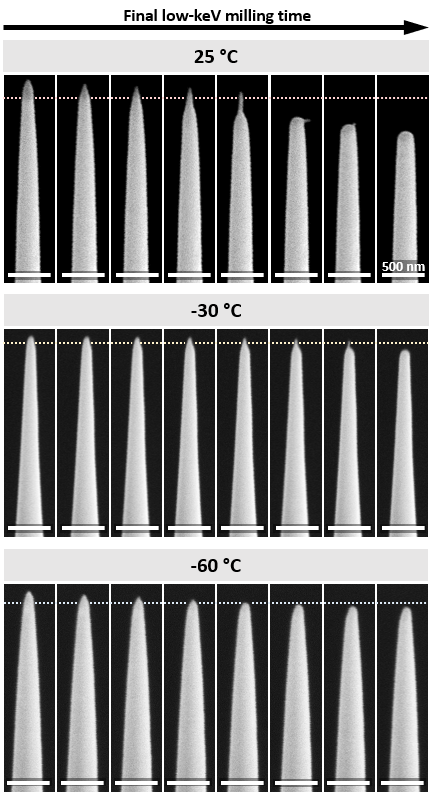


FESEM SE screen‑grabs from during the final low‑keV APT‑specimen milling step utilising 5 keV ion accelerating voltage and 16 pA FIB current. The panels show the specimen geometry evolutions for approximately every 1.5 seconds. The dashed lines mark the respective Pt – Sn interfaces. The specimen evolution during the final milling step is shown at 25 °C, ‑30 °C and ‑60 °C. The respectively last panels show the final APT‑specimens for each milling temperature. Scale bar of 500 nm is valid for all images.

### **Supplementary Figure 6: 3D visualisation of Ga‑concentration in the APT‑specimens from annular milling.**


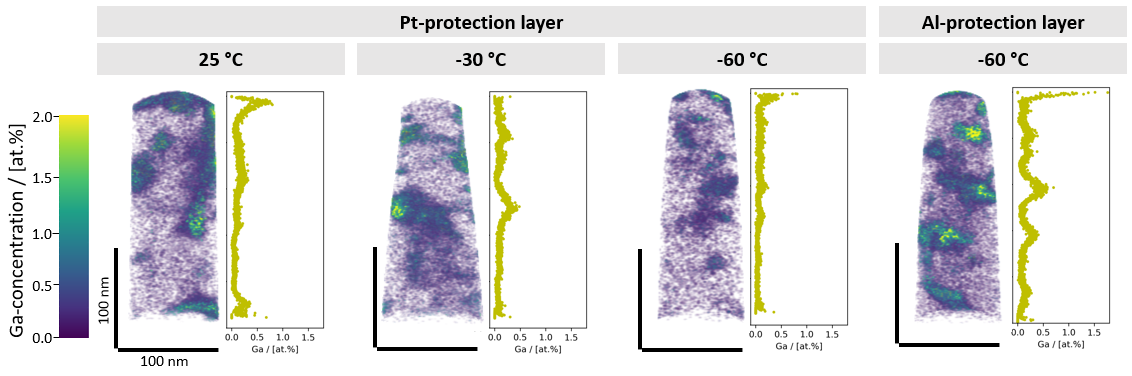


3D Ga‑concentration distributions in the reconstructed APT‑specimens that are milled at 25,  ‑30 and  ‑60°C utilising a Pt‑protection layer, as well as a specimen milled at ‑60°C utilising an Al‑protection layer. The respective Ga‑concentration profiles along the direction of evaporation. The Ga‑concentration distributions are scaled from 0.0 to 2.0 at.% Ga. Scale bars of 100 nm are valid for all images.

### **Supplementary Figure 7: Rotated 3D visualisation of Ga‑concentration distribution in the APT‑specimens.**


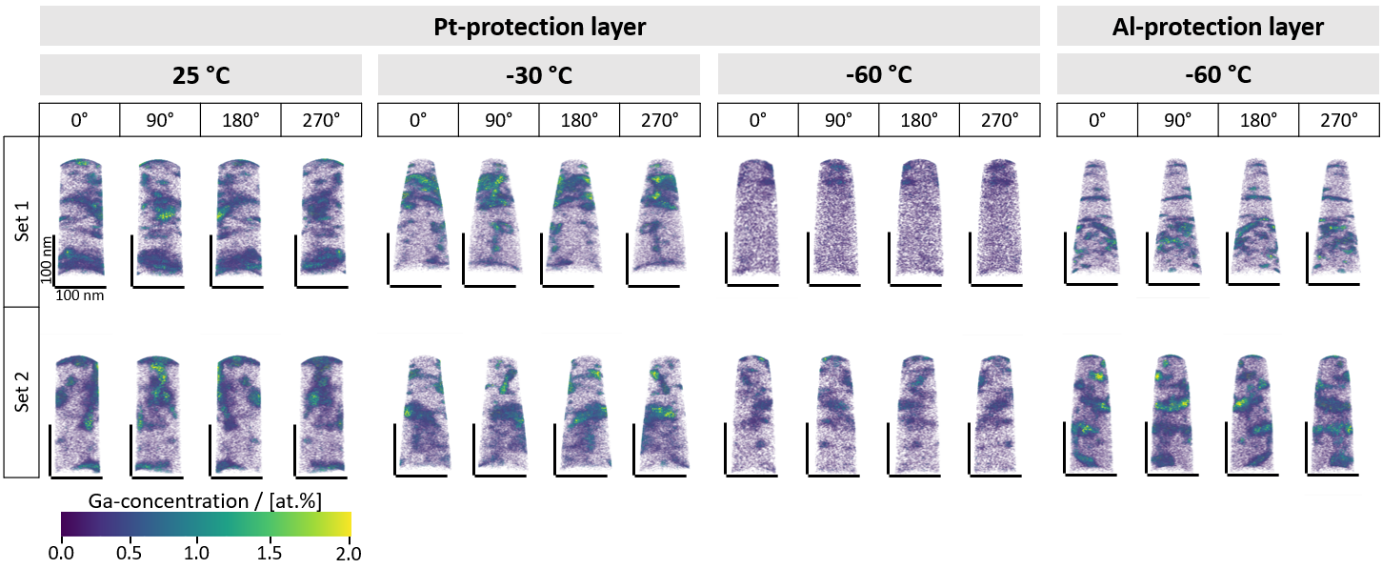


3D Ga‑concentration distributions in both sets of reconstructed APT‑specimens milled at 25,  ‑30 and  ‑60°C utilising a Pt‑protection layer, as well as a specimen milled at ‑60°C utilising an Al‑protection layer rotated 0, 90, 180 and 270° around the direction of evaporation to fully present the 3D volume. The Ga‑concentration distributions are scaled from 0.0 to 2.0 at.% Ga. Scale bars of 100 nm are valid for all images.

### **Supplementary Figure 8: Exemplary APT mass spectra and detector hit maps of the specimens prepared at 25, ‑30 and ‑60 °C, respectively.**


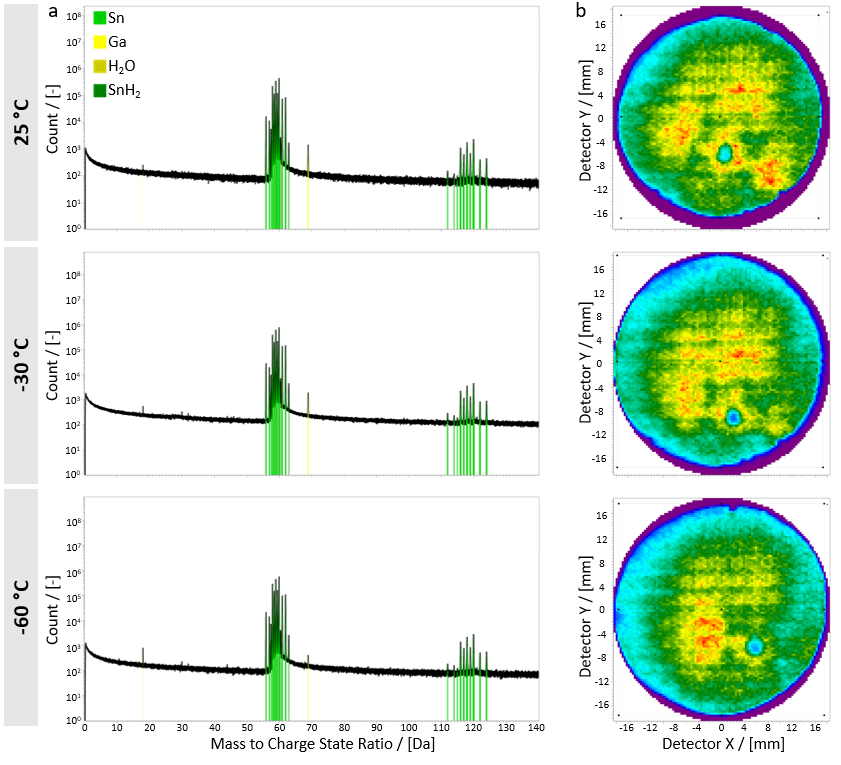


**a** Exemplary APT mass spectra of specimens prepared at 25, ‑30 and ‑60 °C, respectively. Peaks corresponding to Sn are shown in bright green, Ga in yellow, H_2_O in dark yellow and SnH_2_ in dark green. It should be noted that there is only one Ga‑peak positioned at 69 Da, because the specimens are prepared with a single‑isotopic Ga‑FIB source. **b** Detector hit maps of the respective APT measurements in **a**. Crystallographic poles can be seen. Although all specimens stem from the same single crystal region in the sample cross‑section, the poles are not located in the exact same places on the detector, which can be caused by slight differences in the specimen alignment during the lift‑out procedures.

References

1. Larson D, Prosa T, Ulfig R et al. (2013) Local Electrode Atom Probe Tomography: A User's Guide. Springer, New York, NY

2. Gault B, Chiaramonti A, Cojocaru-Mirédin O et al. (2021) Atom probe tomography. Nat Rev Methods Primers 1. https://doi.org/10.1038/s43586-021-00047-w

3. Miller MK, Russell KF (2007) Atom probe specimen preparation with a dual beam SEM/FIB miller. Ultramicroscopy 107:761–766. https://doi.org/10.1016/j.ultramic.2007.02.023

4. Lefebvre-Ulrikson W, Vurpillot F, Sauvage X (2016) Atom Probe Tomography: Put theory into practice. Elsevier

5. Miller MK, Russell KF, Thompson GB (2005) Strategies for fabricating atom probe specimens with a dual beam FIB. Ultramicroscopy 102:287–298. https://doi.org/10.1016/j.ultramic.2004.10.011
